# Nigerian women’s modern contraceptive use: evidence from NDHS 2018

**DOI:** 10.1530/RAF-23-0063

**Published:** 2024-06-10

**Authors:** Ibrahim Banaru Abubakar, Hafsat Banaru Abubakar

**Affiliations:** 1 Department of Family Medicine, Ahmadu Bello University Teaching Hospital, Zaria, Nigeria; 2 Department of Family Medicine, Abubakar Tafawa Balewa University Teaching Hospital, Bauchi, Nigeria

**Keywords:** contraceptive prevalence, family planning, Nigeria, reproductive health

## Abstract

Nigeria has the largest population in Africa, a high fertility rate, and unmet needs for family planning. Family planning is a key strategy for sustainable development. A good knowledge of factors that determine contraceptive uptake is imperative for policy formulation. A nationally representative secondary dataset of 41,821 women aged 15–49 years who participated in the 2018 Nigeria Demographic and Health Survey was analyzed. Multivariate logistic regression was used to examine the association between various factors and the current use of modern contraceptives. The respondents’ average age was 35.9 ± 7.9 years. Overall, contraceptive prevalence was 16.6% for traditional methods and 12.2% for modern methods. Predictors of an increase in modern contraception use were age 40–44 (aOR = 1.07, 95% CI: 0.75–1.53); being a working-class woman (aOR = 1.15, 95% CI: 0.99–1.33); living in an urban area (aOR = 1.14, 95% CI: 0.97–1.33); living in the South West (aOR = 1.36, 95% CI: 1.03–1.79); increasing wealth (aOR = 0.78, 95% CI: 0.66–0.93); and health insurance (aOR = 1.22, 95% CI: 0.89–1.68). Couple dynamics influencing modern contraceptive use were joint decision (aOR = 2.16, 95% CI: 1.81–2.59), self-decision on health care (aOR = 1.34, 95% CI: 1.06–1.70), and earning more than a partner (aOR = 1.14, 95% CI: 0.78–1.66). There are significant variations in contraceptive uptake attributable to socioeconomic and political inequalities, requiring a holistic approach to mitigate barriers and improve contraceptive uptake.

**Lay summary:**

This study examined modern contraceptive use among 41,821 Nigerian women aged 15–49 using data from the 2018 Nigeria Demographic and Health Survey. The average age of the participants was 36 years. The findings showed that 12.2% of women used modern contraceptive methods, while 16.6% used traditional methods. Factors influencing modern contraceptive use included being aged 40–44, being employed, living in urban areas, residing in the South West region, having higher wealth, and having health insurance. Couple dynamics also played a role, with joint decision-making, self-decision on health care, and higher earnings than a partner being associated with increased contraceptive use. The study highlights the importance of addressing socioeconomic and political disparities to improve access to and use of contraceptives for Nigerian women, ultimately contributing to sustainable development.

## Introduction

The use of modern contraception among women of childbearing age is an important aspect of reproductive health because it has the potential to improve maternal and child health outcomes, reduce poverty, and promote gender equality (Li & Rimon 2018). Despite their benefits, however, the use of modern contraception remains low in many developing countries, including Nigeria. In Nigeria, successive demographic and health surveys show only a modest improvement in the use of modern contraception (NPC & ICF 2019, Abubakar 2021).

Several studies have been conducted to investigate the factors influencing contraceptive use in Nigeria. Among the major barriers to the use of modern contraceptives are cultural and religious attitudes, a lack of access to health-care services, a lack of information and education about the benefits of contraception, and poverty (Okonofua 2014, Akamike *et al.* 2020, Anyatonwu *et al.* 2023). Furthermore, low levels of female education, a strong preference for large families, and a lack of support from male partners have all been found to be barriers to modern contraceptive use (Johnson 2017, Alo *et al.* 2020).

Several interventions have been proposed and implemented to address Nigeria’s low contraceptive use, including increasing access to family planning services, improving information and education on the benefits of contraception, and strengthening community-based programs (Hounton *et al.* 2015, Hutchinson *et al.* 2021).

For example, the Nigerian government has launched several programs aimed at increasing access to family planning services, such as the United States-funded Expanded Social Marketing Project (ESMP), which provides a variety of modern contraceptives at a low cost (USAID 2018).

In Nigeria, various types of modern contraceptives are available, including hormonal methods (oral contraceptive pills, injections, and implants) (Anate *et al.* 2021), barrier methods (condoms and diaphragms) (Anate *et al.* 2021), intrauterine devices (IUDs) (Uhuo *et al.* 2020), and fertility awareness-based methods (natural family planning) (Uhuo *et al.* 2020). Hormonal methods are the most commonly used contraceptives among Nigerian women, with 12.3% of women using them. However, access to these methods is still limited, and knowledge about contraceptive options remains inadequate (Uhuo *et al.* 2020). IUD utilization is low in Nigeria, with only 4.7% of women using them. This is due to the fear of side effects, the fear of infertility, and the lack of access to health-care services (Uhuo *et al.* 2020). Barrier methods, such as condoms and diaphragms, are also underutilized, with only 6.7% of women using them. Cultural and religious beliefs discourage their use, and access to these methods is limited (Uhuo *et al.* 2020).

Fertility awareness-based methods, such as natural family planning, are utilized by a small percentage of women in Nigeria, with only 2.1% using them (Uhuo *et al.* 2020). The low utilization of these methods is due to a lack of knowledge, training, and limited access to health-care services. The Nigerian government has taken steps to increase access to these methods. For example, the National Family Planning Program was launched in 2018 with the goal of providing women with increased access to family planning services and information (NPC & ICF 2019). Additionally, the government has also partnered with international organizations and nongovernmental organizations to increase access to modern contraceptives through community-based distribution (Riley *et al.* 2018). In addition to increasing access to modern contraceptives, efforts have also been made to address the cultural and religious barriers to their utilization, as it is established that religious leaders can play a critical role in promoting the use of modern contraceptives by dispelling myths and misconceptions about their use (Adedini *et al.* 2018). The goal of this study is to determine the prevalence of contraceptive use in Nigeria and to investigate the factors that influence contraceptive use in a nationally representative survey.

## Materials and methods

Nigeria, with a population of about 200 million people, comprises 36 states and the Federal Capital Territory, which are grouped into six geopolitical zones (World Bank 2023). The Nigeria Demographic and Health Survey (NDHS) 2018 was conducted by the National Population Commission (aka NPC) with financial and technical support from various organizations and is the sixth and the latest in a series of demographic and health surveys conducted in Nigeria since 1990 (NPC & ICF 2019). The NDHS 2018 sample is nationally representative and covers the entire population residing in noninstitutional dwelling units in the country. The sample was selected through a two-stage cluster sampling design, designed to provide population and health indicator estimates at the national, zonal, and state levels. The study population consisted of women of childbearing age (15–49 years) who were residents of both urban and rural areas in Nigeria. This study used secondary data from the NDHS 2018, extracted from the individual women’s records of the NDHS. The focus of the study was on the subset of data related to family planning, with a particular emphasis on modern contraceptive methods. The data were analyzed using descriptive statistics and multivariate logistic regression to determine the relationship between various factors and the use of modern contraceptives. The level of significance was set at 0.05. Permission was obtained from the data originator, who had previously obtained informed consent from the study participants. This study aimed to contribute to the understanding of the factors influencing modern contraceptive use among Nigerian women of childbearing age and inform evidence-based family planning policies and programs in the country. Data cleaning and preparation were carried out to ensure that the data was accurate and consistent for analysis. Descriptive statistics were used to summarize the demographic characteristics of the study population and the prevalence of modern contraceptive use. Multivariate logistic regression was used to examine the relationship between various factors and the use of modern contraceptives while controlling for potential confounding variables. The factors considered in the regression analysis included the educational level of respondents and their spouses, the wealth index, place of residence, age, and region of the country.

## Results

The study analyzed 41,821 women’s records who responded to the women’s questionnaire during the 2018 NDHS study period, which took place from August 14 to December 29, 2018. The NDHS 2018 applied appropriate survey weighting techniques to make the sample representative of the Nigerian population. Out of the total, 69% of the women had never used a contraceptive, while 31% had either previously used a contraceptive or were currently using one. Specifically, 12% of women reported current use of a modern contraceptive, 4% reported current use of a nonmodern contraceptive, 9% reported previous use of a contraceptive before their last birth, and 5% reported previous use of a contraceptive since their last birth (Table 1). The highest representation of women in the sample was aged 25–29 (28.1%) and 30–34 (22.8%), while the lowest was 45–49 (2.6%) and 15–19 (4.3%). The prevalence of modern contraceptive use was highest among women aged 35–39 (14.7%) and lowest among those with no formal education (4%) (Table 2). Women who were never married had the highest prevalence of modern contraceptive use (17.1%), followed by the currently married (12%), and formerly married (9.3%). The prevalence of modern contraceptive use increased with higher wealth class, with the highest among the richest class (26.6%) and lowest among the poorest class (3.7%). Contraceptive usage was higher among women who resided in urban areas (18.9%) and women who had health insurance (24.7%) than rural residents (8.0%) and those with no health insurance (11.9%). Contraceptive usage was highest among women from the Yoruba group (25.1%) and the Southern regions (South West – 25.5%, South South – 18.4%, and South East – 15.8%) (Table 3). The highest prevalence of modern contraceptive use was among women whose partners had a professional job (21.1%) and who wanted no more children (20.9%). The highest prevalence of contraceptive use was among women who jointly decided on how to spend their earnings (20.6%) and those who took decisions on their health care with their partners (18.5%).

**Table 1:** Pattern of contraceptive use among women.

	Weighted proportion
Pattern of contraceptive use	
Currently using a modern method	12.2
Currently using a nonmodern method	4.4
Not currently using any method, but:	
Used before last birth	9.4
Used since last birth	5.3
Never used a contraceptive	68.7
Study outcome (modern contraceptive use)	
Yes – currently using	12.2
No – not currently using	87.8

**Table 2:** Sociodemographic information of participants.

	Proportion in sample	Modern contraceptive use	
		No	Yes
Age (years) of respondents			
15–19	4.3	95.8	4.2
20–24	19.5	91.6	8.4
25–29	28.1	87.1	12.9
30–34	22.8	86.3	13.7
35–39	15.9	85.3	14.7
40–44	6.8	85.9	14.1
45–49	2.6	89.0	11.0
Educational attainment			
None	46.4	95.8	4.2
Primary	14.9	87.2	12.8
Secondary	30.5	80.1	19.9
Higher	8.2	72.7	27.3
Partners’ educational attainment			
None	37.4	96.1	3.9
Primary	14.1	89.0	11.0
Secondary	33.8	82.3	17.7
Higher	14.7	77.2	22.8
Marital status			
Never married	4.4	82.9	17.1
Currently married	92.6	88.0	12.0
Formerly married	2.9	90.7	9.3
Wealth index			
Poorest	22.1	96.3	3.7
Poorer	22.8	94.1	5.9
Middle	20.6	88.6	11.4
Richer	18.3	81.8	18.2
Richest	16.2	73.4	26.6
Woman currently working			
No	32.4	91.8	8.2
Yes	67.6	86.0	14.0

**Table 3:** Sociodemographic information of participants.

	Proportion in sample	Modern contraceptive use	
		No	Yes
Place of residence			
Urban	38.5	81.1	18.9
Rural	61.5	92.0	8.0
Region			
North Central	13.5	85.5	14.5
North East	18.2	91.9	8.1
North West	36.7	93.8	6.2
South East	10.0	84.2	15.8
South South	8.7	81.6	18.4
South West	12.9	74.5	25.5
Ethnicity			
Hausa	37.8	94.3	5.7
Fulani	8.3	93.3	6.7
Igbo	12.7	81.8	18.2
Yoruba	11.0	74.9	25.1
Others	30.3	85.5	14.5
Number of living children			
None	1,0	99.3	0.7
One	13.9	90.9	9.1
Two	21.0	87.3	12.7
Three	18.3	85.9	14.1
Four or more	45.7	87.7	12.3
Health insurance			
Not insured	97.9	88.1	11.9
Insured	2.1	75.3	24.7
Husband/partner’s age			
Below 30	9.5	90.3	9.7
30–39	41.1	86.2	13.8
40–49	32.8	87.8	12.2
50–59	12.2	89.1	10.9
60+	4.4	92.5	7.5

The results showed that women aged 15–19 years were less likely to use modern contraceptives compared to women aged 45–49 years (OR = 0.36), while women aged 25–29 years and above were more likely to use them compared to the oldest group. Unmarried women were more likely to use modern contraceptives compared to married women (OR = 1.51), while formerly married women were less likely (OR = 0.75) (Table 4). Women with lower educational levels were less likely to use modern contraceptives, with no formal education having the lowest odds (OR = 0.12). Women with jobs were more likely to use modern contraceptives (OR = 1.15), as were women from urban areas compared to those from rural areas (OR = 1.14). Women from South West Nigeria were four times more likely to use modern contraceptives than women from the North West region (OR = 3.90). Women from all ethnic groups except Hausa were more likely to use modern contraceptives. Women in the lower wealth group were less likely to use modern contraceptives compared to women in the richest group (aOR = 0.32 for poorest class). Women with health insurance were more likely to use modern contraceptives than those without (OR = 1.22). Women who make joint decisions on contraceptive usage with their partners were more likely to use modern contraceptives (aOR = 2.16). Women with partners with no formal education levels were less likely to use modern contraceptives (aOR = 0.25). Women with more children were more likely to use modern contraceptives (OR = 0.04 for women with no child and OR = 0.61 for two children). Overall predictors of increase in modern contraception use were, age 40–44 (aOR = 1.07, 95% CI: 0.75–1.53); being a working-class woman (aOR = 1.15, 95% CI: 0.99–1.33); living in an urban area (aOR = 1.14, 95% CI: 0.97–1.33); living in South West (aOR = 1.36, 95% CI: 1.03–1.79); increasing wealth (aOR = 0.78, 95% CI: 0.66–0.93) and health insurance (aOR = 1.22, 95% CI: 0.89–1.68). Couple dynamics influencing modern contraceptive use were, joint decision (aOR = 2.16, 95% CI: 1.81–2.59); self-decision on health care (aOR = 1.34, 95% CI: 1.06–1.70) and earning more than partner (aOR = 1.14, 95% CI: 0.78–1.66) (Table 5).

**Table 4:** Logistic regression on sociodemographic factors associated with use of modern contraceptives among women.

	Un. OR	95% CI	*P*	Adj. OR	95% CI	*P*
Wealth index						
Poorest	0.11	0.08–0.13	<0.001	0.32	0.24–0.42	<0.001
Poorer	0.17	0.14–0.21	<0.001	0.42	0.33–0.54	<0.001
Middle	0.35	0.30–0.42	<0.001	0.60	0.50–0.73	<0.001
Richer	0.61	0.53–0.71	<0.001	0.78	0.66–0.93	0.004
Richest	1			1		
Number of living children						
None	0.05	0.02–0.15	<0.001	0.04	0.01–0.13	<0.001
One	0.71	0.61–0.83	<0.001	0.42	0.34–0.52	<0.001
Two	1.03	0.90–1.18	0.670	0.61	0.51–0.73	<0.001
Three	1.16	1.02–1.33	0.027	0.74	0.63–0.86	<0.001
Four or more	1			1		
Health insurance						
Not insured	1			1		
Insured	2.44	1.76–3.38	<0.001	1.22	0.89–1.68	0.220

Un. OR, unadjusted odds ratio; adj. OR, adjusted OR.

**Table 5:** Logistic regression on male involvement and couple dynamics associated with use of modern contraceptives among women.

	Un. OR	95% CI	*P*	Adj. OR	95% CI	*P*
Decision maker on usage of contraceptive						
Mainly woman	1			1		
Joint decision – woman and partner	2.51	2.17–2.90	<0.001	2.16	1.81–2.59	<0.001
Partner or other persons	0.83	0.67–1.03	0.092	0.99	0.77–1.29	0.960
Partners’ educational attainment						
None	0.14	0.11–0.17	<0.001	0.25	0.18–0.34	<0.001
Primary	0.42	0.35–0.50	<0.001	0.49	0.39–0.63	<0.001
Secondary	0.73	0.63–0.84	<0.001	0.78	0.65–0.95	0.014
Higher	1			1		
Person who decides how to spend woman’s earnings						
Woman and partner	1			1		
Woman majorly	0.56	0.47–0.66	<0.001	0.92	0.75–1.13	0.436
Partner and others	0.45	0.34–0.60	<0.001	0.84	0.61–1.17	0.313
Person who decides woman’s health care						
Woman and partner	1			1		
Woman majorly	1.01	0.83–1.25	0.896	1.34	1.06–1.70	0.016
Partner and others	0.41	0.35–0.46	<0.001	0.89	0.74–1.08	0.234
Living with partner						
Living together	1			1		
Partner staying elsewhere	0.99	0.81–1.22	0.943	0.66	0.51–0.86	0.002

Un. OR, unadjusted odds ratio; Adj. OR, adjusted OR.

## Discussion

The results of this study highlight the disparity in contraceptive use among women in Nigeria. About 69% of the women surveyed had never used a contraceptive, with only 12% reporting current use of a modern contraceptive. The highest representation of women in the sample was aged 25–29 and 30–34, while the lowest was 45–49 and 15–19. The prevalence of modern contraceptive use was highest among women aged 35–39 and lowest among those with no formal education.

Unmarried women had the highest prevalence of modern contraceptive use, followed by the currently married and formerly married. The results also showed that women with higher levels of education and job status were more likely to use modern contraceptives. This aligns with previous research which highlights the importance of education and employment in increasing women’s access to and use of contraception (Krenn *et al.* 2014, WHO 2019). Women from urban areas and those with health insurance were also more likely to use modern contraceptives.

In terms of ethnic and regional differences, women from the Yoruba group and the Southern regions had the highest prevalence of modern contraceptive use. Women from South West Nigeria were four times more likely to use modern contraceptives than women from the North West region. This highlights the importance of addressing regional disparities in access to and utilization of reproductive health services (Okigbo et al. 2017).

Couple dynamics also played a role in contraceptive use, with women who made joint decisions on contraceptive usage with their partners being more likely to use modern contraceptives. This highlights the importance of involving men in reproductive health decision-making and promoting gender equality in partnerships (Tokhi *et al.* 2018, United Nations 2019). Women with partners with no formal education levels were less likely to use modern contraceptives, which highlights the need for increased education and awareness for both women and men.

### Regional and urban–rural disparities

The regional variations identified in this study echo similar patterns consistently observed across Nigeria. Studies reported a recurring observation that individuals in the Southern regions and urban areas are more likely to adopt modern contraceptive services than their counterparts in Northern and rural areas (Johnson 2017; Wang & Cao 2019). This emphasizes the need for targeted interventions to address specific challenges unique to different geographical areas.

### Educational attainment

The inverse relationship between educational attainment and contraceptive use is a recurring theme. This finding resonates with studies conducted in Nigeria by Idowu *et al.* (2020) as well as in other African countries like Emina *et al.* (2014). These studies collectively underscore the role of education as a pivotal determinant of contraceptive practices.

### Ethnic and cultural influences

The ethnic and cultural variations in contraceptive use, particularly the higher prevalence among the Yoruba group, align with findings from studies such as Ajayi *et al.* (2018) in South West Nigeria. Cultural norms and values continue to shape reproductive health behaviors, necessitating culturally sensitive interventions.

### Age and marital status

The age and marital status differentials identified in this study resonate with global trends. Studies conducted in other African countries, such as Durowade *et al.* (2017) and Hellwing *et al.* (2023), have similarly reported age and marital status as crucial determinants of contraceptive practices, emphasizing the need for nuanced interventions targeting specific age and marital cohorts.

### Gender dynamics

The role of couple dynamics in contraceptive decision-making, as illuminated in this study, is consistent with the findings of Blackstone & Iwelunmor (2017) in Nigeria. They found, as evidenced from NDHS 2013, that involving men in reproductive health decisions positively influences contraceptive use, emphasizing the importance of promoting gender equality in reproductive health programs.

### Health insurance and socioeconomic status

The positive association between health insurance, higher socioeconomic status, and contraceptive use echoes findings in studies conducted in other developing countries (Ekholuenetale *et al.* 2022). Access to health insurance emerges as a key facilitator, highlighting the interconnectedness of socioeconomic factors and reproductive health outcomes.

### Longitudinal changes

Longitudinal studies, such as those conducted by Wang & Cao (2019), have tracked changes in contraceptive prevalence over time, utilizing NDHS 2003–2013, offering insights into evolving patterns. Considering the temporal limitations of NDHS 2018, it is imperative to incorporate findings from studies examining contraceptive trends to discern shifts and inform current policy decisions.

Overall, this study found that increasing age, job status, wealth, health insurance, and joint decision-making with partners were factors associated with an increase in modern contraception use. It is important to consider these factors when developing and implementing policies and programs aimed at increasing contraceptive access and utilization in Nigeria.

In conclusion, the results of this study show that a large proportion of women in Nigeria do not use contraceptives. There are disparities in contraceptive use among women based on their age, marital status, education, employment, wealth, health insurance, and regional and ethnic background. Couple dynamics also play a role in contraceptive use, with joint decision-making with partners being positively associated with modern contraception use. Addressing these disparities and promoting gender equality in partnerships is essential for increasing contraceptive access and utilization in Nigeria.

## Declaration of interest

The authors declare that there is no conflict of interest that could be perceived as prejudicing the impartiality of the study reported.

## Funding

This work did not receive any specific grant from any funding agency in the public, commercial, or not-for-profit sector.

## Author contribution statement

IBA conceived the idea, extracted data and did analysis, and discussion. HBA did the background literature, discussion, and conclusion.
